# Modulating Microbial Materials - Engineering Bacterial Cellulose with Synthetic Biology

**DOI:** 10.1021/acssynbio.4c00615

**Published:** 2024-11-07

**Authors:** Koray Malcı, Ivy S. Li, Natasha Kisseroudis, Tom Ellis

**Affiliations:** † Department of Bioengineering, Imperial College London, London SW7 2AZ, U.K.; ‡ Imperial College Centre for Synthetic Biology, Imperial College London, London SW7 2AZ, U.K.; § Department of Life Sciences, Imperial College London, London SW7 2AZ, U.K.

**Keywords:** bacterial cellulose, biomaterial, engineered living materials, synthetic biology, *Komagataeibacter*, 3D bioprinting

## Abstract

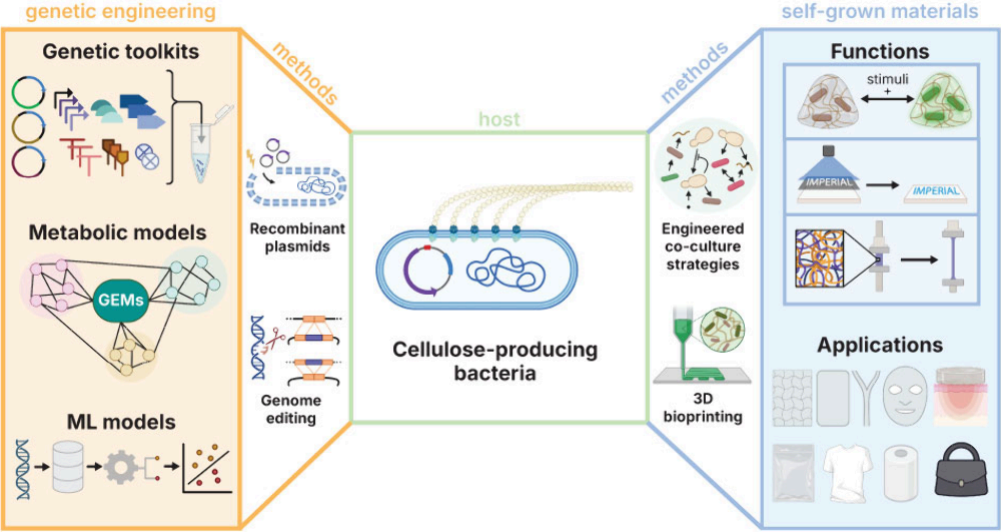

The fusion of synthetic biology and materials science offers exciting opportunities to produce sustainable materials that can perform programmed biological functions such as sensing and responding or enhance material properties through biological means. Bacterial cellulose (BC) is a unique material for this challenge due to its high-performance material properties and ease of production from culturable microbes. Research in the past decade has focused on expanding the benefits and applications of BC through many approaches. Here, we explore how the current landscape of BC-based biomaterials is being shaped by progress in synthetic biology. As well as discussing how it can aid production of more BC and BC with tailored material properties, we place special emphasis on the potential of using BC for engineered living materials (ELMs); materials of a biological nature designed to carry out specific tasks. We also explore the role of 3D bioprinting being used for BC-based ELMs and highlight specific opportunities that this can bring. As synthetic biology continues to advance, it will drive further innovation in BC-based materials and ELMs, enabling many new applications that can help address problems in the modern world, in both biomedicine and many other application fields.

## Introduction

Over the past two decades, a significant fraction of synthetic biology (SynBio) research has concentrated on developing the sustainable production of products traditionally sourced from petrochemicals. And more recently, there has been a particular emphasis on using the nature of biology to impart programmability and tunability in making and designing materials.^1,2^

While various organisms, including bacteria,^3^ fungi,^4^ and plants,^5^ can be employed for making materials via biopolymer synthesis, SynBio research predominantly works with microbes, especially easy to culture and engineerable bacteria and yeasts. Their use is driven not only by their relative ease of handling but also by the availability of DNA toolkits and computational resources for genetic manipulation. Using modular genetic engineering, SynBio researchers have the opportunity to control and modify the material production from microbes. This can occur at the polymer scale, for example to tune the physicochemical properties, such as molecular weight, monomer composition, 3D structure, and chain length.^6^ Or across the macroscale to develop tailored biomaterials with desired thermochemical and mechanical characteristics suitable for diverse applications, from medical research to bioremediation.^7^ The use of SynBio to modify microbes that can produce materials, take functional roles, or incorporate functional elements within materials is a core part of the emerging topic of Engineered Living Materials (ELMs).

**Figure 1:**
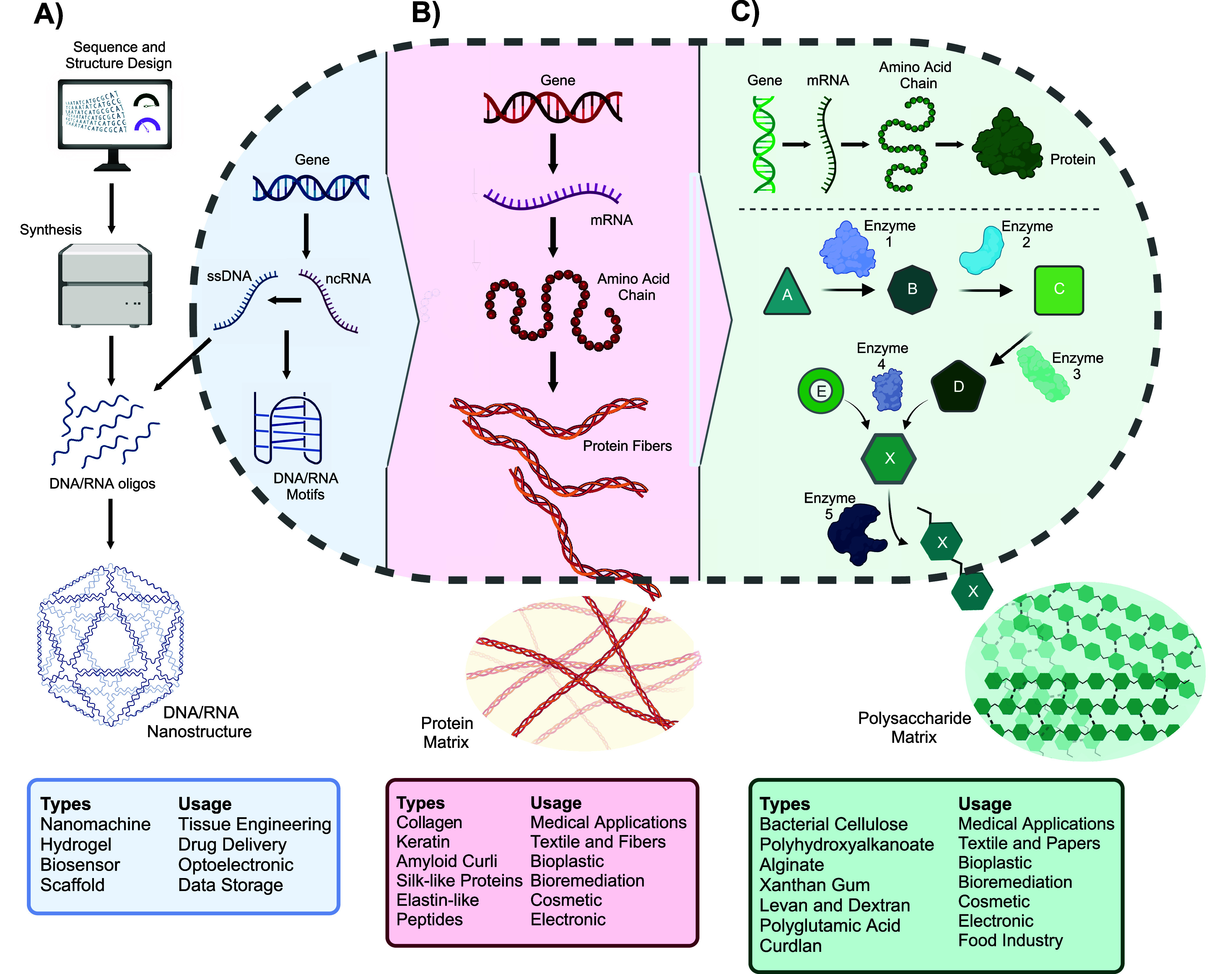
Biopolymer Synthesis Hierarchy. **(A)** DNA/RNA Polymers: DNA or RNA nanostructures can be computationally designed and synthesized *in vitro* for applications like drug delivery. Single-stranded DNAs (ssDNAs) can also be produced *in vivo* from noncoding RNA, either as building blocks for *in vitro* production or as *in vivo* DNA motifs. **(B)** Protein-Based Polymers: To produce polymers like collagen or amyloid curli, the genetic code in DNA is expressed, controlling the protein’s 3D structure, function, and expression rate. Protein expression involves complex transcription and translation processes, making it more complex than DNA-based polymer production. Proteins can be stored intracellularly or secreted. **(C)** Polysaccharide-Based Polymers (e.g., Bacterial Cellulose): These polymers are synthesized through complex metabolic pathways involving multiple enzymes and regulators. Production and feature alteration require fine-tuning of specific steps or a holistic system-level approach. Like proteins, these biopolymers can be stored or secreted.

Polymer-producing bacteria are especially important for ELMs, as they can be engineered to produce biomaterials with enhanced utility, such as self-repair, and sensing and responding capabilities.^2,8^ Where engineering of polymer-producers is difficult, co-culturing these bacteria with microbes that can be engineered, for example to secrete enzymes, presents another option for material functionalization.^9^ Bacteria inherently produce a wide range of polymers, not just proteins, nucleic acids, and polysaccharides, but also polyamides, polyesters, and even bioceramics, each serving distinct functions in their physiological processes.^10^ As well as storing genetic information and serving as energy reservoirs, bacteria use biopolymers to form protective layers around cells, contributing to biofilm formation and creating extracellular structures to shield bacterial communities from their environment^11^ (Figure 1). The derivatives of these polymers have addressed diverse societal and environmental applications across fields such as therapeutic treatments, manufacturing biological bricks, and the bioremediation of pollutants.^12,13^

Among bacterially made polysaccharides, the extracellular polysaccharide bacterial cellulose (BC) has gained significant attention for its abundance, biocompatibility, high water-holding capacity, and permeability.^14^ These characteristics make BC a good candidate for developing novel functionalized materials, suitable for a range of uses from encapsulating bioactive molecules through to being a bulk polymer for fabricating sustainable materials.^9^ To date, nonmodel acetic acid bacteria have been the main focus for BC production platforms.^8^ But more recently genetic engineering of these bacteria has been considered for producing more advanced materials, especially in the domain ELMs.^15^

In this review, we cover the current landscape of how SynBio approaches are being used to make BC-based materials that are both nonliving and living when in use. We explore the potentials offered by BC-based materials while evaluating the current bottlenecks and challenges in their development. Through this, we provide insights for future research and innovation in BC-based biomaterial design.

## Bacterial Cellulose Materials

Bacterial cellulose (BC) is chemically identical to the cellulose found in plant cell walls, consisting of linear polymer made of glucose monomers linked by β-1,4 glycosidic bonds.^16,17^ At the macroscale, BC is visibly produced as a hydrogel-like pellicle at the air–liquid interface of static cultures of Gram-negative aerobic bacteria.^18,19^ The structure of BC is hierarchical and self-assembles from secreted high-aspect-ratio cellulose polymers. These polymers agglomerate into nanofibrils and microfibrils through intra- and intermolecular interactions.^20,21^ The result of the supramolecular interactions is a material that is characterized by its high purity, degree of polymerization, crystallinity, and remarkable mechanical strength, as well as its higher water retention, permeability, porosity, and biocompatibility.^22−27^

**Figure 2:**
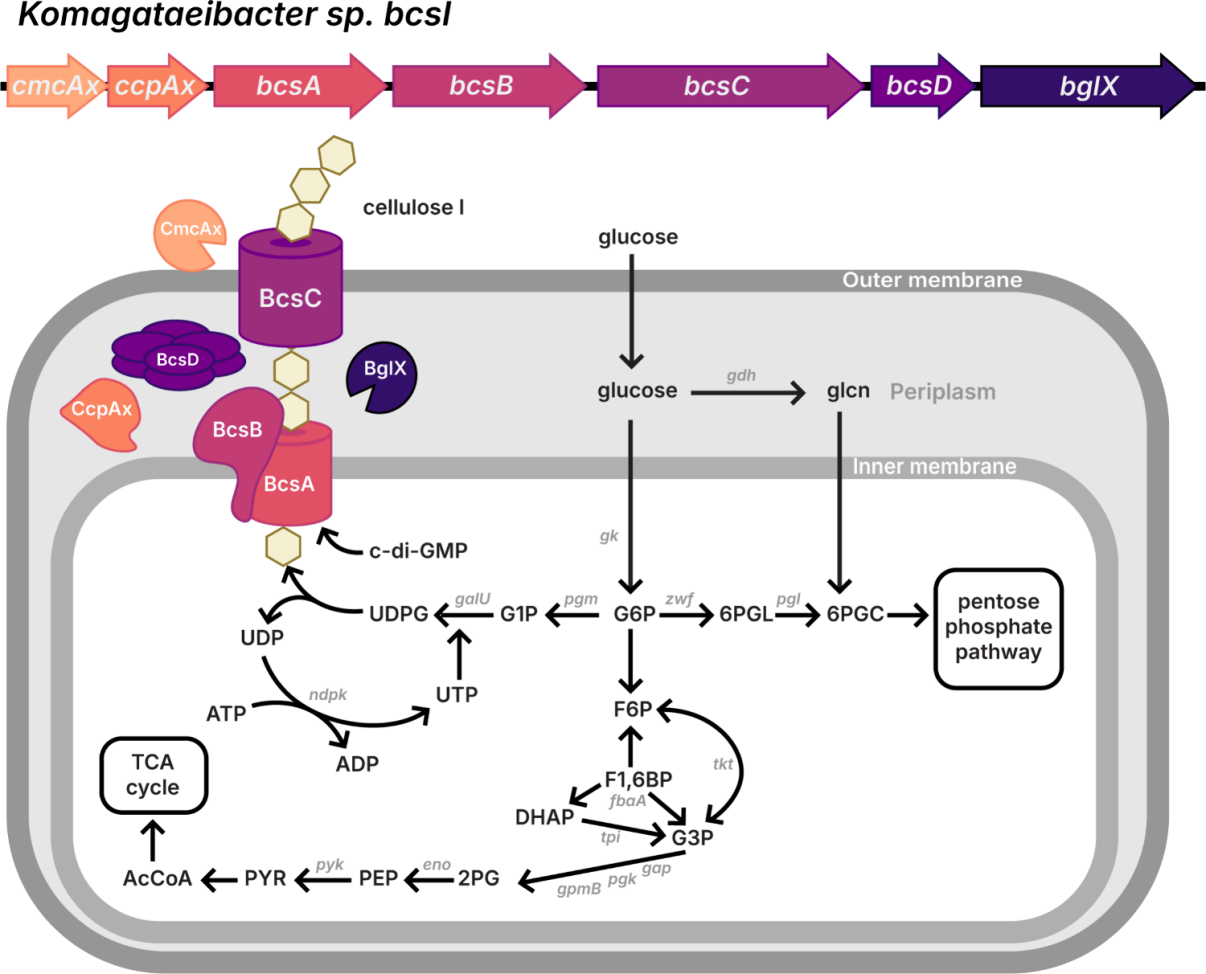
Overview of BC synthesis in *Komagataeibacter spp*. Relevant *bcs* cellulose synthase operon genes *bcsABCD* and the accessory genes *cmcAx, ccpA*, and *bglX*, and their respective proteins are highlighted in color. The complete *bcsI* (type I) operon is shown here for simplicity, though several other copies of the *bcs* operon, typically modified, exist throughout the *Komagataeibacter* species chromosome. Cellulose is synthesized from glucose, which is converted into UDP-glucose before being added onto to the reducing end of the glucan chain (shown as yellow hexagons) at the inner membrane and then exported out of the bacterial cell. Various modifications to the cellulose chain occur in the periplasm and extracellularly. Enzyme abbreviations from top to bottom: *gdh* (glucose dehydrogenase), *gk* (glucose kinase), *galU* (UTP-glucose-1-phosphate uridylyltransferase), *pgm* (phosphoglucomutase), *zwf* (glucose 6-phosphate 1-dehydrogenase), *pgl* (6-phosphogluconolactonase), *ndpk* (nucleoside diphosphate kinase), *tkt* (transketolase), *fbaA* (fructose bisphosphate adolase A), *tpi* (triosephosphate isomerase), pyk (pyruvate kinase), *eno* (enolase), *gpmB* (2,3-bisphosphoglycerate-independent phosphoglycerate mutase B), *pgk* (phosphoglycerate kinase), and *gap* (glyceraldehyde 3-phosphate dehydrogenase). Metabolite abbreviations from top to bottom: glcn (gluconate), c-di-GMP (cyclic diguanylate), UDP (uridine diphosphate), UDPG (uridine diphosphate glucose), G1P (glucose 1-phosphate), G6P (glucose 6-phosphate), 6PGL (6-phosphogluconolactone), 6PGC (6-phosphogluconate), ATP (adenosine triphosphate), ADP (adenosine diphosphate), UTP (uridine triphosphate), F6P (fructose 6-phosphate), F1,6BP (fructose-1,6-diphosphate), DHAP (dihydroxyacetone phosphate), G3P (glyceraldehyde-3-phosphate), AcCoA (acetyl coenzyme A), PYR (pyruvate), PEP (phosphoenol pyruvate), and 2PG (2-phosphoglyceric acid).

The exceptional mechanical properties of BC arise from the nanoscale self-assembly of the individual cellulose nanofibrils. The synthesis of BC is primarily driven by the bacterial cellulose synthase (*bcs*) operon which shows variable genetic organizations across species.^28^ In many cellulose-producing bacteria including *Komagataeibacter* species the *bcs* operon consists of four subunits, BcsA, BcsB, BcsC, and BcsD as the bcsI (type I) cellulose synthase operon (Figure 2).^28,29^ The cellulose synthase complex, also known as the terminal complex, intriguingly forms linear pores at the longitudinal axis of the bacteria.^30−32^ Although BcsA and BcsB catalytic subunits can show cellulose synthesis activity *in vitro*, all subunits are essential for efficient *in vivo* cellulose synthesis, packaging, crystallization, and exportation.^28,33^ The synthesis process initiates at the periplasmic membrane, as shown in Figure 2, where BcsA and BcsB form a heterodimer as a catalytic core capable of facilitating a condensation reaction between UDP-glucose and the reducing end of the glucan chain.^34−36^ This catalytic activity is dependent on the binding of the global allosteric regulator cyclic-di-GMP to a PilZ regulatory domain on BcsA.^37,38^ Following synthesis, the cellulose chain is translocated through the periplasmic membrane and secreted extracellularly via the β-sheet barrel BcsC in the outer membrane.^39^^40^ The BcsD subunit forms an octamer that can bind up to four glycan chains and is putatively located in the periplasm.^40,41^ Mutagenesis and overexpression of BcsD suggest that BcsD is associated with cellulose crystallinity.^32,42^

Accessory genes located in the flanking regions of the cellulose synthase operon play crucial roles in cellulose synthesis, particularly in its regulation and packaging (Figure 2). In *Gluconacetobacter* and *Komagataeibacter* lineages, the cellulose-complementing protein A (CcpAx—also called BcsH) is essential for cellulose production. CcpAx influences the expression levels of BcsB and BcsC, and also interacts with BcsD. Additionally, it has been shown to facilitate the organization of glucan chains into crystalline cellulose ribbons.^43,44^ The *cmcAx* gene, also known as *bcsZ*, encodes carboxymethyl cellulase, an endo-β-1,4-glucanase that selectively degrades amorphous and disordered cellulose but is ineffective against crystalline forms. By breaking down the tangled cellulose, it facilitates the normal crystallization process during cellulose synthesis, which can also lead to an increase in overall cellulose production.^45^*BglX* is another gene located in close proximity to the cellulose synthase subunits and encodes a β-glucosidase, which is thought to have a similar function to that of CmcAx. It was also reported that *bglX*-deficient *K. xylinus* strains produced dramatically lower cellulose with more than 80% decrease in the production.^46^

Despite significant advances in research of the cellulose synthase proteins, the complete interaction of the terminal complex between all four cellulose synthase proteins, their accessory proteins, and the multiple genomic copies of the operon are not yet fully understood. This gap in knowledge presents challenges in establishing a comprehensive link between genotype and phenotype, particularly in relation to the properties of the produced material. From a synthetic biologist’s perspective, BC synthesis has been expertly evolved and conserved in BC producers, efficiently utilizing glucose to produce high yields of an exopolysaccharide with remarkable material properties—the foundation for further customization.

## BC Producers and Their Genetic and Computational Tools

BC synthesis was first documented in the 19th century by A. J. Brown, who observed that *Acetobacter xylinum* (now reclassified as *Komagataeibacter xylinus*(47)) could produce cellulose during aerobic fermentation when supplied with glucose.^19^ Since then, numerous BC-producing bacteria have been identified, with most belonging to Gram-negative species, as shown in Figure S1, although Gram-positive BC producers like *Sarcina ventriculi* also exist. Gram-negative BC producers include species from the genera *Acetobacter*, *Gluconacetobacter*, *Komagataeibacter*, *Agrobacterium*, *Rhizobium*, *Pseudomonas*, *Salmonella*, *Azotobacter*, *Achromobacter*, and *Alcaligenes*.^48,49^ While some species synthesize BC for specific biological functions, such as flocculation, plant attachment, or maintaining an aerobic environment, others produce BC only under particular environmental conditions.^50^

Research on BC has predominantly centered on *Komagataeibacter*, which are recognized for their proficient extracellular BC synthesis. *K. xylinum*, *K. hansenii* (also known as *Novacetimonas hansenii*(51)), *K. sucrofermentans*, and *K. rhaeticus* have emerged as putative model organisms for BC production and found use in commercial applications. These organisms have been experimentally explored in terms of their metabolism, BC biosynthesis pathways, and genetic content.^52,53^ Consequently, SynBio tools and methods have been developed to engineer these organisms for tailored and functionalized BC synthesis and specific applications.

### SynBio Genetic Tools for BC-Producing Bacteria

**Figure 3:**
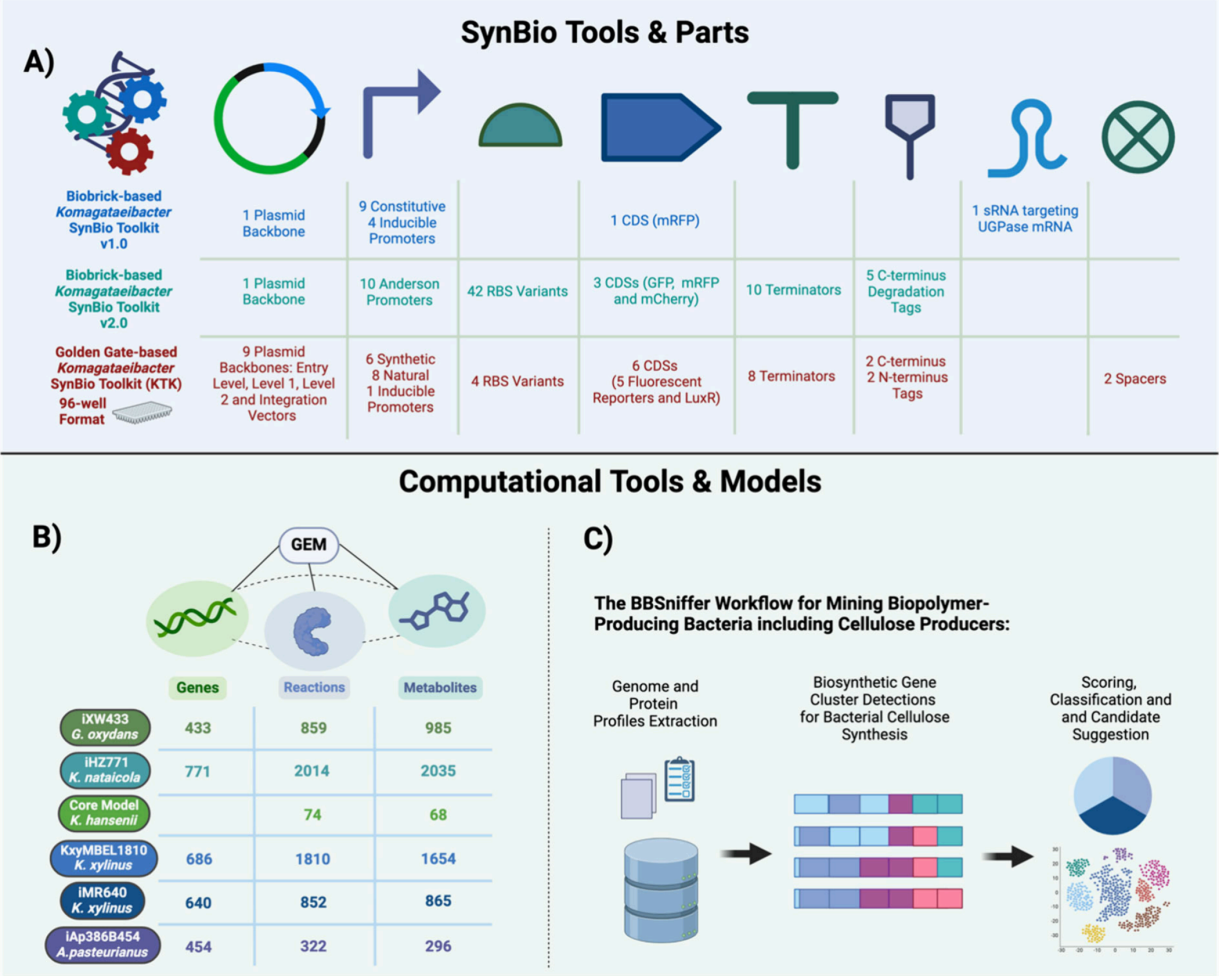
SynBio and computational tools for engineering BC producers. **(A)** The genetic toolkits developed for *Komagataeibacter*. The first toolkit is based on Biobrick assembly with constitutive and inducible promoters and a small RNA (sRNA) targeting UGPase mRNA. The second version of the Biobrick-based toolkit, offering a wide array of promoters, RBSs, coding sequences (CDSs), terminators, and protein tags, tested and characterized in *K. xylinus, K. hansenii*, and *K. rhaeticus*. The Golden Gate-based *Komagataeibacter* SynBio toolkit (KTK) enables the hierarchical assembly of transcription units and multicassette plasmids, available as a cost-effective 96-well plate on Addgene. **(B)** Genome-scale metabolic models (GEMs) for BC-producing species provide mathematical models that link genes, reactions, and metabolites to accelerate strain design through simulations and predictions of genomic modifications. **(C)** BBSniffer, a computational workflow that recommends BC producers under specific conditions such as growth environments or the availability of SynBio tools. The parts provided via Addgene are shown in the toolkits.

Before synthetic biology, the earliest attempts at genetic manipulation to improve BC production began two decades ago with *K. xylinus* with the engineered heterologous expression of sucrose synthase.^27,54^ However, the most significant advance for SynBio was the development of the first modular genetic toolkit for BC-producing bacteria, in this case a series of plasmids and DNA parts for engineering *K. rhaeticus* (Figure 3).^55^ This toolkit included nine minimal constitutive promoters (taken from a set used in *Escherichia coli*), as well as anhydrotetracycline (aTc) and *N*-acyl-homoserine lactone (AHL) inducible promoters, an inducible small RNA (sRNA) construct targeting UGPase mRNA. To use these parts in the bacteria, a plasmid with BioBrick-compatible multiple cloning sites, pSEVA331Bb, was developed.^55^ This toolkit’s capabilities were demonstrated by showing the functionalization and patterning of BC with a red fluorescent protein (mRFP1) and was shared widely via the nonprofit Addgene. Later, this toolkit was expanded with additional modular DNA parts and characterized by others across two other species of *Komagataeibacter*: *K. xylinus*, *K. hansenii*.^56^ Six more minimal constitutive promoters, an arabinose-inducible promoter, five protein degradation tags, 42 ribosomal binding sites (RBS), and 10 terminators (five synthetic and five natural) were introduced, further advancing *Komagataeibacter* SynBio applications.^56^ This expanded toolkit was used to engineer cells to produce a chitin-cellulose copolymer and was also made available via Addgene.^57^

Since its introduction, Golden Gate Cloning has become the primary DNA assembly method used by the SynBio community^58−60^ due to its ability to facilitate precise and high-throughput DNA assembly in a standardized manner.^61^ Thus, a significant advancement for *Komagataeibacter* SynBio was the introduction of the Golden Gate-based *Komagataeibacter* Toolkit (KTK), which includes hierarchical assembly plasmid with various antibiotic selection markers, and a selection of DNA parts including promoters, RBSs, terminators, neutral spacers, and N-terminal and C-terminal peptide tags.^62,63^ The KTK system is especially well-suited to assemble multigene cassettes and was used in *K. rhaeticus* to construct a six-gene operon alongside a LuxR transcriptional unit, enabling the cells to be programmed to extrude curli amyloid fibers in response to AHL induction.^62^ A further demonstration used multiple expression cassettes to secrete elastin-like polypeptide (ELP) and β-lactamase (BLA) proteins through a Type VII secretion system.

Genome editing methods, particularly CRISPR-based tools, are extensively employed for strain engineering in many model organisms.^64,65^ The use of CRISPR technology in BC-producing bacteria has also been reported, with the noncutting CRISPR interference (CRISPRi) system applied to downregulate the cellulase synthase operon in *K. hansenii*, targeting the *acsAB* coding sequence.^56^ This resulted in a more than 2-fold reduction in expression of this gene and a 15% decrease in cellulose production.^56^ CRISPRi was also used in *K. xylinus* to modulate the expression of the *galU* gene, which encodes UGPase, a key enzyme controlling carbon flux between BC synthesis and the pentose phosphate pathway.^66^ By altering *galU* expression levels, the structural features of BC, such as porosity and crystallinity, were regulated.^66^

These studies demonstrate the potential of CRISPR technology for genome editing and BC manipulation in *Komagataeibacter* species. While CRISPRi relies on a catalytically inactive Cas9 (dCas9) protein,^67^ the CRISPR/Cas9 system employs an active Cas9 that can also facilitate marker-free genomic deletions or integrations. Although previous studies have reported the use of λ-Red-mediated homology-directed repair (HDR) and suicide vectors for marker deletion by flanked FRT sites and Flp recombinase, the efficiency of DNA repair mechanisms, such as HDR and nonhomologous end joining (NHEJ), in *Komagataeibacter* species remains largely unexplored. These mechanisms are crucial for repairing double-stranded DNA breaks induced by Cas9 activity.^62,68−71^ Also, further research is needed to identify and validate effective guide RNA (gRNA) sequences, particularly the crRNA binding sites, for CRISPR applications in *Komagataeibacter*. Additionally, work is needed to integrate *Komagataeibacter* genomes into widely used computational gRNA prediction tools, such as CRISPOR, which currently includes 1,151 genomes and provides species-specific off-target effects and efficiency scores for crRNA sequences.^72^ This could further advance CRISPR-based manipulation in BC producers.

### Computational Tools and Models

Predictability is a fundamental engineering principle in synthetic biology. As a result, genome-scale metabolic models (GEMs), which provide computational associations among genes, proteins, and reactions across an entire living system, have become an invaluable tool for *in silico* simulations and predictions. Such models have been developed for numerous organisms and have been used for a variety of applications.^73−76^ With the advances in “omics” technologies, genome and system-level data have also been generated and integrated for key BC producers, enabling the reconstruction of GEMs for these species (Figure 3D).

The first GEM for a BC producer was reconstructed a decade ago for *Gluconobacter oxidans*, incorporating 433 genes, 859 reactions, and 985 metabolites. This model, GEM iXW433, was used for *in silico* simulations to predict essential genes and reactions.^77^ In *Komagataeibacter nataicola*, the GEM iHZ771, comprising 771 genes, 2,035 metabolites, and 2,014 reactions, was reconstructed to identify potential genomic targets for enhancing BC production.^78^ Simulations using a core model of *K. hansenii* accurately predicted growth under various media and carbon sources, consistent with experimental data.^79^ The GEM KxyMBEL1810, developed for *K. xylinus*, connected 686 genes, 1,810 reactions, and 1,654 metabolites.^69^ It was utilized to predict essential genes involved in BC biosynthesis and potential overexpression targets to boost BC production. Indeed, by expressing heterologous *pgi* and *gnd* genes in *K. xylinus*, BC synthesis was improved by 115.8% compared to the parent strain, based on *in silico* predictions from this GEM.^69^ Another GEM for *K. xylinus*, iMR640, demonstrated 93.7% accuracy when compared to experimental BC production data.^80^ For *Acetobacter pasteurianus*, the GEM iAp386B454 was reconstructed with 454 genes, 322 reactions, and 296 metabolites across two cellular compartments.^81^ Additionally, two derivatives of this model were created that focus on its core metabolism and energy production.^82^

More recently, the bioinformatics tool Bacteria Biopolymer Sniffer (BBSniffer) was developed to facilitate genetic mining of biopolymer producers, including BC-producing bacteria. This tool is based on specific constraints, such as growth conditions or the availability of SynBio tools for bacteria, and can accelerate biopolymer design and ELM development.^83^ The BBSniffer workflow begins by extracting the genome and protein profiles, followed by protein sequence alignment using Clustal Omega, which generates related hidden Markov model profiles. It then employs a modified version of antiSMASH to target and detect biopolymer biosynthetic gene clusters (BGCs).^84^ The software then builds an internal bacterial database, classifying organisms as pathogens, industrial strains, or other nonpathogens. Finally, it constructs a distance-based phylogenetic tree using a reference species to suggest candidate organisms.^83^ For BC production under aerobic conditions at 28–30 °C and pH 3.5–8.0 in DSMZ medium 1044, and with the availability of the CRISPRi tool, the BBSniffer workflow identified a *K. rhaeticus* strain and *Zymomonas mobiliz* strains as promising candidates for BC production when *K. xylinus* was used as the reference species.^83^

The availability of the genetic and computational tools described above provides a robust foundation for designing customized BC-producing strains, not only for enhanced cellulose production but also for a wide range of applications, such as expressing recombinant proteins or engineering BC with specific structural features.^85^ Importantly as the genetic toolkits developed adhere to widely established modular DNA standards (BioBricks and Golden Gate), research groups using these can add new parts to the toolkits or bring in DNA parts from other toolkits and can easily share these among different groups, broadening the potential genetic engineering in BC-producing species. GEMs also offer invaluable system-level insights into a host organism’s metabolism. *In silico* simulations using GEMs can predict promising genomic modifications for specific purposes or simulate the organism’s behavior under various growth conditions. Regular updates to the GEMs for BC producers, similar to those for model organisms like yeast and *E. coli*, could enhance their potential, accuracy, and adoption.^86,87^ However, achieving this requires a more established computational biology community working on BC producers.

## Genetic and Metabolic Engineering for BC Production and Functionalization

### Engineering BC Producers

Genetic manipulations in BC producers have primarily focused on enhancing the BC production yield, enabling BC synthesis on alternative media, and improving BC properties with new functions or structures. Due to the need for large-scale and cost-effective BC production, efforts to optimize BC synthesis began decades ago. Inspired by higher plants that utilize sucrose synthase to increase UDP-glucose concentrations for cellulose production, Nakai et al. (1998) expressed a mutant version of mung bean sucrose synthase in *K. xylinus*, achieving more than a 2-fold increase in BC production compared to the wild-type strain.^54^ Subsequent studies of note include work that integrated the *lacZ* gene into the *K. xylinus* genome, enabling cellulose production in lactose-containing media, such as whey, and research that added constitutive expression of the *Vitreoscilla* hemoglobin (VHb) gene in *K. xylinus* to enhance intracellular dissolved oxygen levels and increase the growth rate and double cellulose production.^68,88^ Further VHb expression work in *K. xylinus* increased BC production by 70% in static culture with lower glucose consumption and by 58.6% under 15% oxygen tension.^89,90^

Metabolic engineering of *K. xylinus* has further improved BC production by establishing and enhancing the glycolysis pathway in this bacterium. This has been achieved by coexpressing the *E. coli* cAMP receptor protein, a transcription factor that positively regulates glucose-metabolizing genes, and the *E. coli* phosphofructokinase enzyme, a key step in glycolysis missing in *K. xylinus*, under control of the pTac promoter.^91^ This engineering approach not only improved BC production yield but also reduced the formation of the byproduct gluconic acid. This engineered strain was successfully employed for large-scale BC production in 30 L fermenters, with the resulting BC being used in the fabrication of cylindrical lithium-ion batteries.^92^ The batteries demonstrated a remarkable performance with 80% capacity retention after 1000 cycles, comparable to commercial equivalents.

Recent studies have continued to focus on enhancing BC production in *K. xylinus* by various strategies, including tuning gene expression with synthetic ribosome binding sites (RBSs), by modifying the BC biosynthesis pathway and/or related pathways, by overexpressing the cellulose synthase operon, or by improving the strain’s ability to cope with oxygen tension by overexpressing aerobic respiration control protein A.^69,93−98^ Additionally, recombinant expression of mannose kinase and phosphomannose isomerase genes from *E. coli* has enabled *K. xylinus* to utilize mannose for BC production, a strategy to increase BC yield.^99^

Although research on the model BC producer *K. xylinus* has dominated the literature, alternative BC producers have also been genetically engineered to enhance BC production. In *K. hansenii*, overexpression of the *motA* and *motB* genes, which may be involved in cell motility by the formation of a proton pump, led to improved BC productivity with thicker cellulose filaments and elongated cellular phenotypes.^100^ Previous studies on *K. xylinus* have demonstrated that disrupting the pyrroloquinoline quinone (PQQ) cofactor-dependent glucose dehydrogenase enzyme, which oxidizes glucose to gluconic acid, can more than double BC production and improve glucose utilization.^101^ This modification could potentially enable the use of glucose-rich waste as a carbon source.^102^ Indeed, in a recent study, knocking out the PQQ-dependent glucose dehydrogenase *gdh* in *K. sucrofermentans* resulted in a more than 2-fold increase in BC production.^103^ A similar study also with *gdh* knockout *K. xylinus* strain also overexpressed glucose transporter gene *gllf* from *Zymononas mobiliz* and native glucose kinase *glk* to increase glucose uptake in the cytoplasm and increase BC production.^71,104,105^

Improving BC production yield or enabling the use of a broader range of cost-effective carbon sources for BC synthesis can be a significant step toward the industrial-scale application of BC. However, considering the challenges associated with engineering BC-producing bacteria and the relatively low value of cellulose compared to other high-value chemicals produced by engineered microorganisms, enhancing BC production through synthetic biology may not be the most efficient strategy for BC utilization.^106^ Indeed, increasing the value of BC by adding additional features and functions could transform it into a high-value material suitable for specific applications, potentially replacing traditional materials that are unsustainable or less cost-effective. To achieve this, BC producers have been engineered to grow functionalized cellulose designed for specific purposes.

Research on modifying BC features has primarily focused on expanding its use in biomedical applications. The first cellulose/chitin copolymer was synthesized using an engineered *K. xylinus* expressing three genes from the UDP-GlcNAc synthesis operon of *Candida albicans*, resulting in a cellulose-based polymer that can be digested by human enzymes, useful as a basis for surgical implants designed to degrade over time.^107^ By simply expressing the curdlan synthase gene from *Agrobacterium sp*. ATCC31749 in *K. xylinus*, a BC/Curdlan composite could be made, giving a BC material with reduced water permeability.^108^ Meanwhile, overexpression of the *motA* and *motB* genes in *K. hansenii* led to a relaxed BC fiber formation, enabling use as a 3D scaffold for chondrocytes in tissue engineering applications, such as cartilage formation.^109^ A BC/hyaluronic acid (HA) copolymer has also been achieved in *K. hansenii*, by expressing the hyaluronan synthase gene from *Pasteurella multocida* ATCC15742 and the UDP-glucose dehydrogenase gene from *Sinorhizobium meliloti* 1021, in both cases with expression controlled by the *trc* promoter with a *lac* operator.^110^

Engineering has also been used for sustainability applications. As an eco-friendly alternative to colored textiles, a tyrosinase gene (*tyr1*) from *Bacillus megaterium* was expressed in *K. rhaeticus* to synthesize eumelanin from l-tyrosine in the presence of oxygen, resulting in a dark black coloration of BC.^111^ This melanated black version of BC was demonstrated as a material that could be used to make wallets and shoes, showcasing its versatility as a textile. Furthermore, using an optogenetic system with a blue-light-sensitive split T7 RNA polymerase (Opto-T7RNAP), this coloration could be extended to be patterned in response to a light projection.^115,116^ Highly detailed, red-patterned BC was produced by using this optogenetic system to express the *mCherry* red fluorescent protein in *K. rhaeticus*.^111^

### Modifying BC through Co-Culturing and Ex-Situ Modification

BC can be modified not only by engineering the BC-producing bacteria but also through the use of additional engineered strains. This can be achieved by co-culturing two organisms together where the non-BC producer modifies the BC or by applying products from a designed organism to the BC to enhance its properties.

An example of this approach used a genetic protein fusion of the hydrophobin BslA from *Bacillus subtilis* with a cellulose binding module (CBM) from *Trichoderma reesei*, with the BslA-CBM fusion protein being recombinantly expressed in *E. coli*.^112^ The crude extract of BslA-CBM was then used to treat dried BC either *ex situ*, through drip coating, or *in situ*, by incorporating it in the growth media as the BC was grown. It was reported that *in situ* incorporation of BslA altered the mechanical properties of BC, producing a stronger and more elastic material, while *ex situ* coating with BslA improved the hydrophobicity of BC, a critical feature for reducing water evaporation within the material.^112^

Another method employed a co-culturing approach to produce BC/hyaluronic acid (HA) composites. In this system, *K. hansenii* was cocultured with an engineered *Lactococcus lactis* strain expressing heterologous *hasABC* genes expressing the key enzymes in HA synthesis pathway, from *Streptococcus zooepidemicus*. This co-culture approach was used in a two-vessel system and under varying initial pH conditions to produce the BC/HA composites.^113,114^

These advances, summarized in Table 1, highlight the growing potential of engineered BC as a versatile material for a wide range of sustainable and specialized applications.

**Table 1:** Genetic Manipulations of BC Producers and Co-Cultured Organisms to Enhance BC Synthesis or Modify BC Properties

Objective	Engineered Organism	Genetic Manipulation	Outcome	Reference
Enhancing BC Production	*K. xylinus*	Expression of mutant mung bean sucrose synthase	Two- to 3-fold increase in BC production	(54)
Lactose utilization	*K. xylinus*	Integration of *lacZ* gene	Enabled cellulose production in lactose-containing media	(68)
Enhancing BC Production	*K. xylinus*	Expression of *Vitreoscilla* hemoglobin gene	Enhanced cellulose production up to 2-fold	(88−90)
Enhancing BC Production and the use of BC in rechargeable batteries	*K. xylinus*	Expression of *E. coli* cAMP receptor protein and phosphofructokinase	Improved BC synthesis yield and reduced gluconic acid formation; produced large scale BC and effective lithium rechargeable battery	(91)
Enhancing BC Production	*K. xylinus*	Modifying *bcsA* gene to avoid IS element insertion to prevent noncellulose-producing mutants	1.7-fold increase in BC production	(94)
Enhancing BC Production	*K. xylinus*	Tuning gene expressions in BC pathway with synthetic RBSs, expressing *pgm*, *galU*, and *ndp* genes in cellulose pathway with a more effective RBS	More than 4-fold increase in BC production in shaking condition with 3.67 g/L	(93)
Enhancing BC Production	*K. xylinus*	Overexpression of *bcsD* genes in cellulose synthesis	More than 3-fold increase in BC production with 6.8 g/L	(95)
Enhancing BC Production	*K. xylinus*	Modifying phosphoenolpyruvate-dependent glucose phosphotransferase system with the expression of *E.coli ptsHIcrr*, *ptsG* and *pfkA* genes with similar expression rates	87% increase in BC production with 7.74 g/L in static culture, longer fiber diameter, lower porosity and higher solid content, crystallinity, tensile strength, and Young’s modulus, eliminated gluconic acid accumulation	(96)
Enhancing BC Production under Increased Oxygen Tension	*K. xylinus*	Overexpression of the fumarate nitrate reduction protein and aerobic respiration control protein A	37% increase in BC production under 40% oxygen tension	(98)
Enhancing BC Production	*K. xylinus*	Heterologous expression of the pTac promoter-driven *pgi* or *gnd* genes from *E.coli* or *C. glutamicum*	115% increase in BC production with 3.15 g/L	(69)
Enhancing BC Production	*K. xylinus*	Overexpression of *bcsABCD* genes in the cellulose synthase operon with arabinose inducible promoter	4-fold increase in BC production with 4.3 ± 0.46 g/L, thicker BC films	(97)
Enhancing BC Production	*K. hansenii*	Overexpression of *motA* and *motB* genes	Thicker cellulose filaments	(100)
Enhancing BC Production	*K. xylinus*	Disruption of PQQ cofactor-dependent glucose dehydrogenase enzyme	More than doubled BC production	(101)
Enhancing BC Production	*K. sucrofermentans*	Knocking out PQQ-dependent glucose dehydrogenase	More than doubled BC production	(103)
Enhancing BC Production	*K. xylinus*	Knocking out PQQ-dependent glucose dehydrogenase and expressing glucose facilitator Glf	30% increase in BC production and glucose utilization	(71)
Mannose utilization	*K. xylinus*	Expression of *E. coli* mannose kinase and phosphomannose isomerase genes	84% increase in BC production in mannose-containing medium	(99)
BC/chitin copolymer production	*K. xylinus*	Expression of three genes from UDP-GlcNAc synthesis operon of *Candida albicans*	More biodegradable cellulose polymer	(107)
BC/Curdlan composite production	*K. xylinus*	Expression of curdlan synthase gene from *Agrobacterium sp*. ATCC31749	Produced BC/Curdlan composites with reduced water permeability	(108)
Developing mammalian cell support	*K. hansenii*	Overexpression of *motA* and *motB* genes	Relaxed BC fiber formation used as 3D scaffold for chondrocytes	(109)
BC/HA copolymer production	*K. xylinus*	Expression of hyaluronan synthase gene and UDP-glucose dehydrogenase gene	Synthesized BC/hyaluronic acid copolymer	(110)
Melanated black BC synthesis	*K. rhaeticus*	Expression of tyrosinase gene from *Bacillus megaterium*	Produced dark black-colored BC for use in textiles and materials	(111)
Optogenetic patterning of BC	*K. rhaeticus*	Expression of mCherry using blue-light-sensitive T7-RNA polymerase system from *E. coli*	Produced red-patterned BC	(111)
Altered mechanical properties of BC	*E.coli*(*in situ*/*ex situ* treatment)	Fusion of BslA with a cellulose-binding module expressed in *E. coli*	Altered mechanical properties of BC, stronger and more elastic material or more hydrophobic BC	(112)
BC/HA copolymer production via cocultivation	*Lactococcus lactis*(coculture)	Co-culturing *K. hansenii* with *Lactococcus lactis* expressing *hasABC* genes from *Streptococcus zooepidemicus*	Produced BC/HA composites	(113,114)

## BC-Based Engineered Living Materials (ELMs)

**Figure 4:**
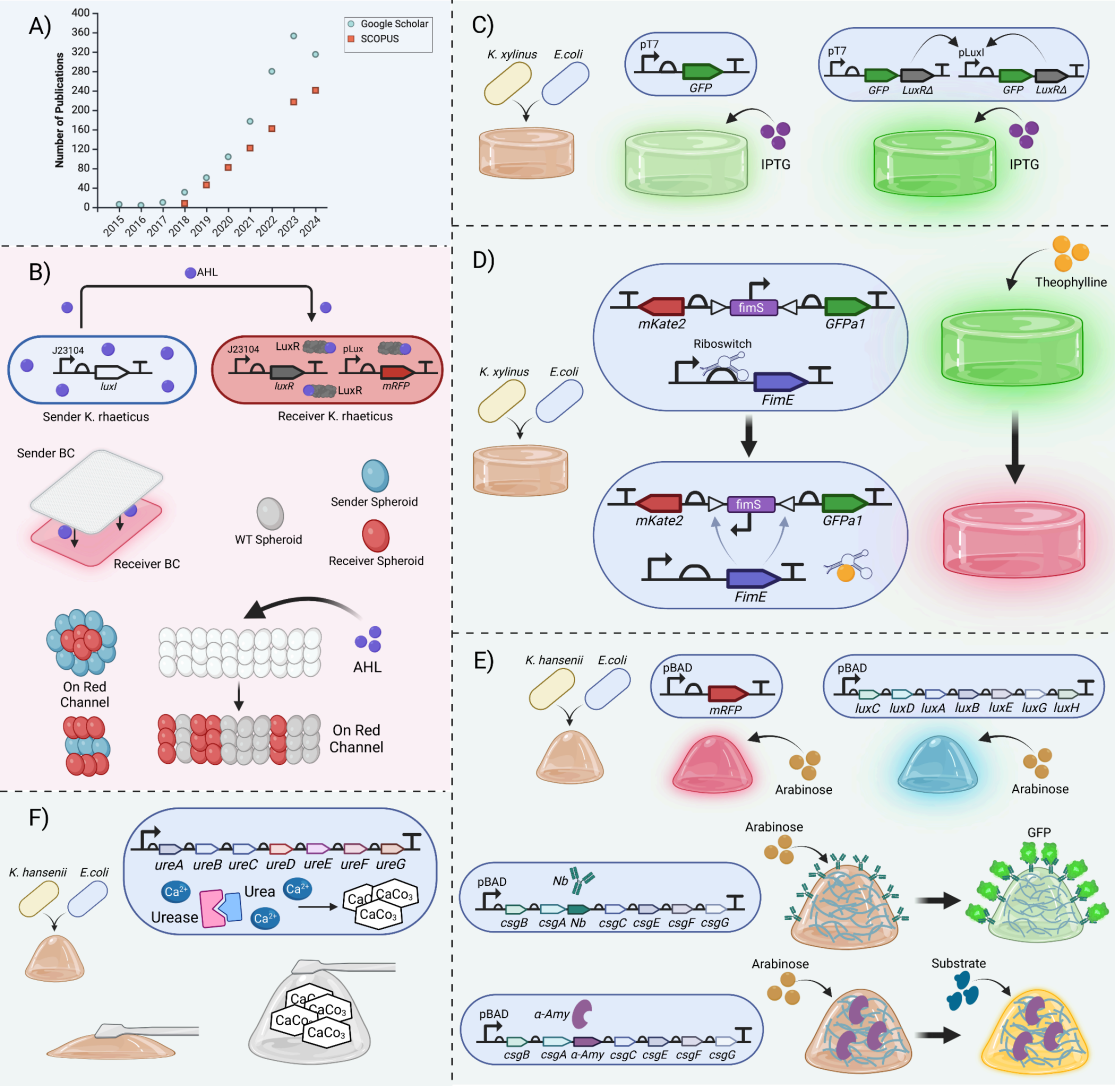
BC-based Engineered Living Systems (ELMs) containing one species of BC producer or bacteria-bacteria consortium for functionalizing ELMs. **(A)** Trends in ELM research: graph shows the number of publications per year containing terms ’engineered living material’ or ’engineered living materials’ over time. Data sourced from Google Scholar and SCOPUS, on 22nd August 2024. **(B)** Sender and Receiver ELMs: *K. rhaeticus* Sender strains secrete AHL, which activates gene expression in Receiver strains by inducing LuxR to activate the pLux promoter, leading to red fluorescence (mRFP) production. This interaction can occur within the same ELM or across two sections of BC. Additionally, BC spheroids can be arranged to produce specific patterns by using Sender and Receiver spheroids. **(C)** IPTG sensor ELM: co-culture of *K. xylinus* and *E. coli* for sensing IPTG. The system uses pT7 promoter to express GFP. This was enhanced by a positive feedback loop where the GFP-LuxRΔ protein amplifies its own expression, resulting in a stronger fluorescent signal. **(D)** Riboswitch-based dual reporter ELM: constructed with *K. xylinus* and *E. coli*, this ELM switches from green fluorescence (GFPa1) to red fluorescence (mKate2) in response to theophylline using a riboswitch-controlled recombinase FimE that inverts the direction of a constitutive promoter on an invertible DNA segment (fimS). **(E)** Chemical-responsive ELMs: developed via co-cultivation of *K*. *hansenii* and *E. coli*, these ELMs respond to arabinose by producing red fluorescence or luminescence. Alternatively, a BC/curli hybrid can also be synthesized with either a GFP-specific nanobody or α-amylase fused to CsgA, enabling GFP sequestration or yellow color production in response to α-amylase substrate. **(F)** Rigid ELM with urease expression: co-cultivated *K. hansenii* and *E. coli* that expresses urease, which degrades urea to produce CaCO_3_ in the presence of calcium ions, significantly increasing the stiffness of the ELM.

Advancing BC-based materials through the development of engineered living materials (ELMs), where living systems are integrated into the material, might represent the most effective and flexible approach to creating a new generation of smart, self-healing, and responsive materials. Since the introduction of the term “smart living material” in 2011, where researchers used the fungus *Penicillium roqueforti* to develop a multilayered living material, there has been a significant surge in ELM research, particularly since 2020 (Figure 4A).^117^ In recent years, many comprehensive reviews have been published, evaluating the advances and future directions of ELMs.^118−124^ A taxonomy for ELMs, categorising them based on features such as the type of organism used, material composition, and dimensionality, has also been proposed.^125^

Fungal mycelium and bacterial polymers like curli or cellulose fibers have been utilized in the development of ELMs, as well as in “hybrid ELMs” where abiotic materials serve as scaffolds for living systems.^123^ BC has significant potential as the bulk material for ELMs as it can provide a support for living systems, including bacteria-bacteria and bacteria-eukaryote cocultures.^126,127^

### Monoculture ELMs with a BC-Producer

A BC producer itself can be engineered to develop a BC-based ELM capable of sensing and responding to environmental signals, as illustrated in Figure 4B. A nice example of this is the adaptation of the Lux quorum-sensing system from *Vibrio fischeri* into *K. rhaeticus* to develop “Sender” and “Receiver” strains for RFP-based material patterning.^128^ In this system, the Sender *K. rhaeticus* strain constitutively expresses the LuxI protein, an *N*-acyl-1-homoserine lactone (AHL) synthase, to produce the signaling molecule AHL. The Receiver strain expresses mRFP under the control of an AHL-inducible promoter, pLux, to report the detection of the chemical signal. This setup enabled cell-to-cell communication and patterning within the ELM, as well as BC pellicle-to-pellicle communication between the Sender and Receiver materials, each containing the engineered strains.^128^ A similar ELM approach was employed using BC spheroids, millimeter-scale rounded BC particles produced under specific shaking conditions.^129,130^ A Receiver spheroid fluoresced red upon contact with a Sender spheroid releasing AHL. This method also could be used as part of a patterning approach, for example using AHL to activate a hidden barcode pattern from strategically placed engineered BC spheroids.^129^^120^

### ELMs with Bacteria-Bacteria Coculture

Taking advantage of well-characterized organisms with extensive genetics tools can significantly enhance the capabilities of ELMs. An early attempt to create an ELM by co-culturing *K. xylinus* with a recombinant *E. coli* strain was conducted to evaluate a BC-based material’s response to inducers.^131^ Initially, an IPTG-inducible GFP expression system in *E. coli* was integrated into the material, followed by the design of a positive genetic feedback loop using *luxR* and pLux promoters in *E. coli* to amplify GFP expression and signaling in response to inducer inputs (Figure 4C).^131^*K. xylinus* was co-cultured with an engineered *E. coli* strain containing a riboswitch-based dual-color reporter system to develop an ELM capable of sensing and responding to target chemicals.^132^ This riboswitch-based sensing system included the recombinase FimE, controlled by a synthetic riboswitch binding to its RBS, and an invertible DNA segment (fimS) containing a constitutive promoter flanked by two fluorescent protein genes. In the absence of the chemical inducer (the asthma drug theophylline), the riboswitch prevented FimE translation, allowing the fimS promoter to express a green fluorescent protein (GFPa1). Upon theophylline addition, FimE translation was initiated, leading to the unidirectional inversion of the fimS segment and the constitutive expression of a red fluorescent protein (mKate2) as demonstrated in Figure 4D.^133^

Researchers successfully achieved a green-to-red fluorescence shift in the ELM in the presence of theophylline.^132^ The fluorescence change in individual cells was imaged by confocal microscope, and the overall fluorescence change in the ELMs was visualized by transilluminator. Additionally, they noted that the BC scaffold, supported with silk fibroin, resulted in a leakage-free material in terms of *E. coli* containment.^132^ This smart approach allowed for the easy monitoring of *E. coli* viability within the material, while the reporter provides a distinct signal upon detecting the target chemical.

In another study, *K. hansenii* was co-cultured with engineered *E. coli* strains to develop a hybrid polymer with BC/curli-based ELM and to produce biomineralized capsules.^134^ Instead of forming the typical BC pellicle at the air interface, researchers engineered a hollow spherical form of BC by growing the co-culture on superhydrophobic powder. In these spherical BC capsules, *K. hansenii* became relatively more dominant at the outer layer, where oxygen was present, then transferring the BC encapsulated cells to another medium containing antibiotic selection marker for the engineered *E. coli* strain. The formation of the co-culture was initially confirmed by inducing mRFP expression in *E. coli* with arabinose.^134^ To further verify the viability of *E. coli* cells within the capsules, researchers expressed the *luxCDABEGH* operon from *Vibrio harveyi* in *E. coli*. This produces 7 proteins that in the presence of ATP produce a luminescence reaction. The detection of a luminescence signal after nutrient depletion confirmed the prolonged viability of *E. coli* cells within the capsule (Figure 4E).^134^

Next, a functional BC/curli structure was developed by expressing an arabinose-inducible curli operon (*csgBACEFG*) in *E. coli*, with either a GFP-specific nanobody or *Bacillus licheniformis* α-amylase fused to the CsgA subunit. This design allowed the curli fibers to sequester GFP when available in the media and produce yellow products when the α-amylase substrate 4-nitrophenyl α-d-maltohexaoside was present (Figure 4E).^134^ Additionally, the urease gene cluster from *Sporosarcina pasteurii* was expressed in *E. coli*, enabling the hydrolysis of urea to produce carbonate ions, which in the presence of calcium ions led to the formation of CaCO_3_, calcium carbonate that can be considered as a key component of bioconcrete. This process significantly increased the stiffness of the ELM capsules (Figure 4F), along with providing them distinct physical characteristics.^134^

### ELMs with Bacteria-Yeast Coculture

**Figure 5:**
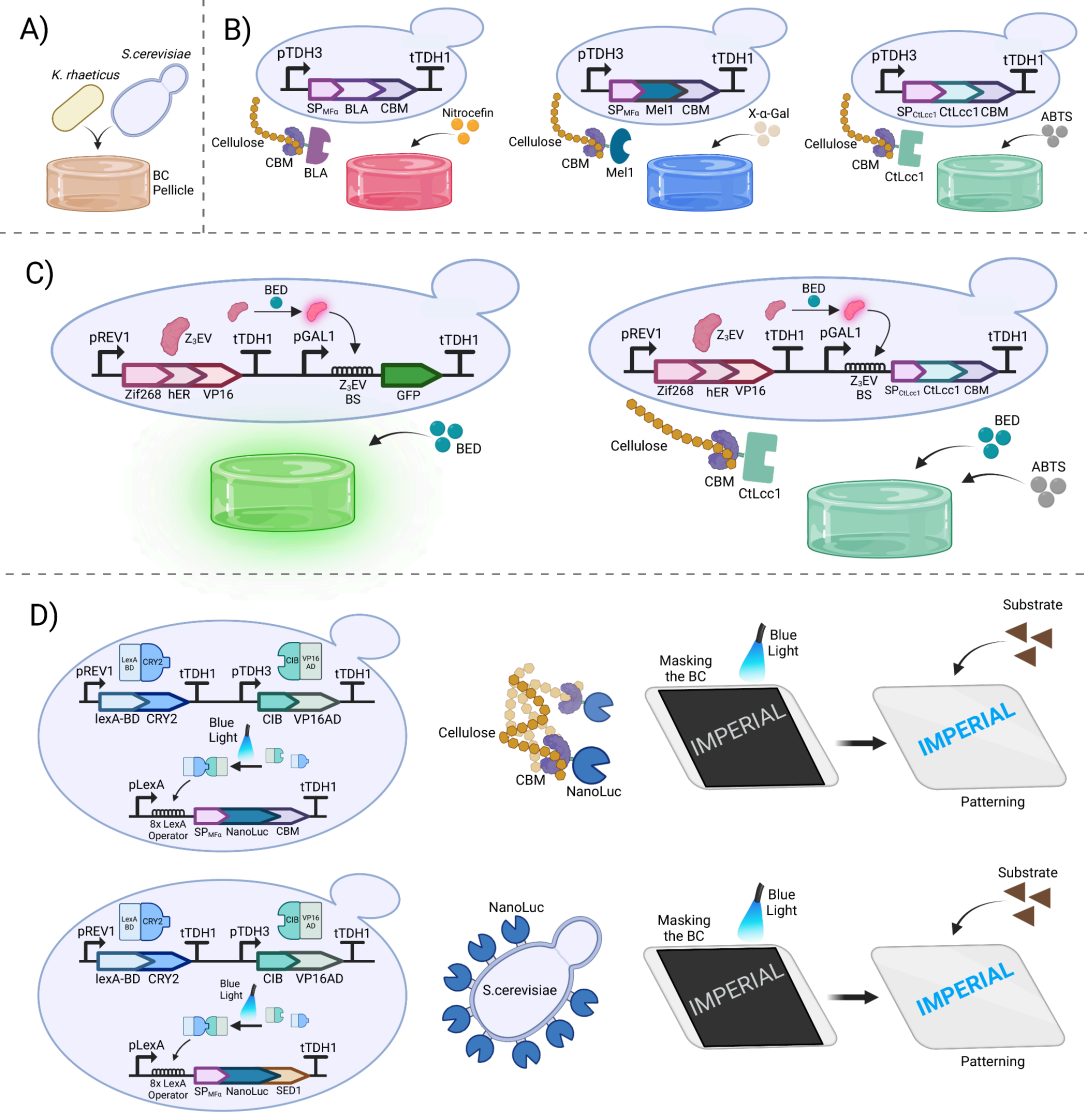
Functionalized ELMs by a coculture of a BC-producer and yeast. **(A)** Syn-SCOBY: a synthetic coculture of *K. rhaeticus* and *S. cerevisiae*, where yeast converts sucrose to glucose for *K. rhaeticus* utilization. **(B)** Functionalization of ELMs with extracellular enzymes: yeast secretes CBM-fused enzymes like β-lactamase (BLA), α-galactosidase (Mel1), and laccase (CtLcc1) to bind cellulose fibers in ELMs, producing specific-colored responses upon substrate addition; red color with BLA and nitrocefin, blue color with Mel1 and X-α-Gal, and dark green color with CtLcc1 and ABTS. MF-α secretion signal peptide (SP_MF-α_) or native secretion signal peptide (SP_CtLcc1_) were used to secrete the fused constructs. **(C)** Estrogen sensor ELM: a human hormone β-estradiol (BED) sensor that activates the Z_3_EV transcription factor in yeast, triggering GFP or CtLcc1-CBM expression, resulting in green fluorescence or dark green color in response to BED. **(D)** Optogenetic patterning: a blue-light-inducible system in ELMs activates CRY2-CIB fusion, binding to LexA operators (8x LexA) in the pLexA promoter to activate NanoLuc expression. Upon substrate addition, a blue pattern forms where a blue light is applied.

A co-culture of *K. rhaeticus* bacteria with the eukaryote *S. cerevisiae*, referred to as a Syn-SCOBY (synthetic symbiotic coculture of bacteria and yeast), was developed to functionalize BC pellicles with engineered yeast strains.^135^ In this system, summarized in Figure 5A, sucrose is used as the carbon source, allowing *K. rhaeticus* to grow on glucose digested from sucrose by the invertase enzyme secreted by yeast. Three engineered yeast strains were used to functionalize the BC pellicles: one secreting β-lactamase (BLA), another secreting α-galactosidase (Mel1), and the third secreting laccase from *Coriolopsis trogii* (CtLcc1). These enzymes were all fused with a cellulose-binding module (CBM) from *Cellulomonas fimi*, to reduce enzyme leakage from the BC pellicles, resulting in higher enzymatic activity due to the increased retention of the enzyme within the material.^135^ Functionalization was demonstrated by the formation of different colors in the Syn-SCOBY-based materials: yellow after the addition of the BLA substrate nitrocefin, blue from X-α-Gal digestion by Mel1, and dark green as a result of ABTS oxidation by CtLcc1.^135^ Cell viability and enzymatic activities were preserved in the BC-based materials grown from Syn-SCOBYs, even after the dehydration and rehydration processes for all three ELMs.^135^

Additionally, a chemically inducible system based on sensing estrogen hormone β-estradiol (BED) was employed. In this system, BED activates a synthetic transcription factor (Z3EV), which then initiates transcription of GFP in yeast via a Z3EV-responsive promoter.^136^ Using yeast with this system in a Syn-SCOBY results in an ELM able to detect BED and produce fluorescent pellicles in response, and remarkably this activity was shown to be present even after over 4 months of storage of the ELM after its production.^135^ The Syn-SCOBY system also provides a yeast-based route into pattern formation in grown BC ELMs. The blue-light activated CRY2–CIB optogenetics system can be used in yeast to trigger expression and secretion of the luciferase reporter enzyme, NanoLuc.^137^ A text-like pattern was created by selectively masking a culture of growing BC, exposing the cells to a projected light source. The addition of NanoLuc’s substrate over the BC material once it is grown then activates the bioluminescence pattern.^135^

## Biofabrication of ELMs Via 3D Printing

The ability to precisely control spatial design by 3D printing is a key advantage in the development of ELMs, as it enables the creation of intricate structures that can mimic or form to natural biological materials, such as human tissues.^138^ When filtered and incorporated into a 3D printing ink comprised of cells and supportive high-viscosity components, BC offers both a sustainable alternative to conventionally used synthetic polymer-based 3D-printing materials and coupled with the ongoing Synbio research the construction of more complex and dynamic BC-based ELMs.^139,140^

For example, this can involve 3D bioprinting BC-producing bacteria in defined spatial arrangements that grow into BC materials with customized geometries.^141^ Schaffner *et al.* (2017) developed a 3D bioprinting system that allows for the digital creation of independent, cell-laden hydrogels, providing complete control over the spatial arrangement and density of cells or microbes within intricate and self-sustaining 3D structures. *K. xylinus* was embedded within a biocompatible hydrogel by incorporating the cells into 3D printing bioink (defined as a cell-containing 3D printing ink^142^) to be printed in the shape of a facial skin scaffold that resembled a doll face.^143^ The scaffold was then incubated for 4–7 days to promote *in situ* cellulose formation by the preloaded bacteria. After cellulose synthesis, the bacteria and ink components were washed away, leaving a BC in the same shape as the 3D printed skin scaffold.^143^ An alternative example of bioink development was demonstrated by Qian *et al.* (2020), who substituted the main bioink component polymers with nanocellulose and yeast cells, combined with poly(ethylene glycol) dimethacrylate and a photoinitiator to photocure the 3D-printed structure. The cell viability after printing matched that of freeze-dried yeast granules, indicating that the 3D printing and photocuring processes did not hamper cell survival.^144^

Cell viability is a significant challenge associated with bioink development in 3D bioprinting, as a suitable bioink must support cell viability while also maintaining viscousness during extrusion printing and gelation of support components. This is a significant challenge, because cells often cannot survive the melting temperatures of the bioink components. One way to address this challenge with Synbio is by engineering cells to be more thermotolerant or loading inks with thermophilic bacteria. Notably *Bacillus subtilis* can survive 20 min incubation at 75 °C making it ideal for an agarose-based ink which melts at 65 °C.^145^

**Figure 6:**
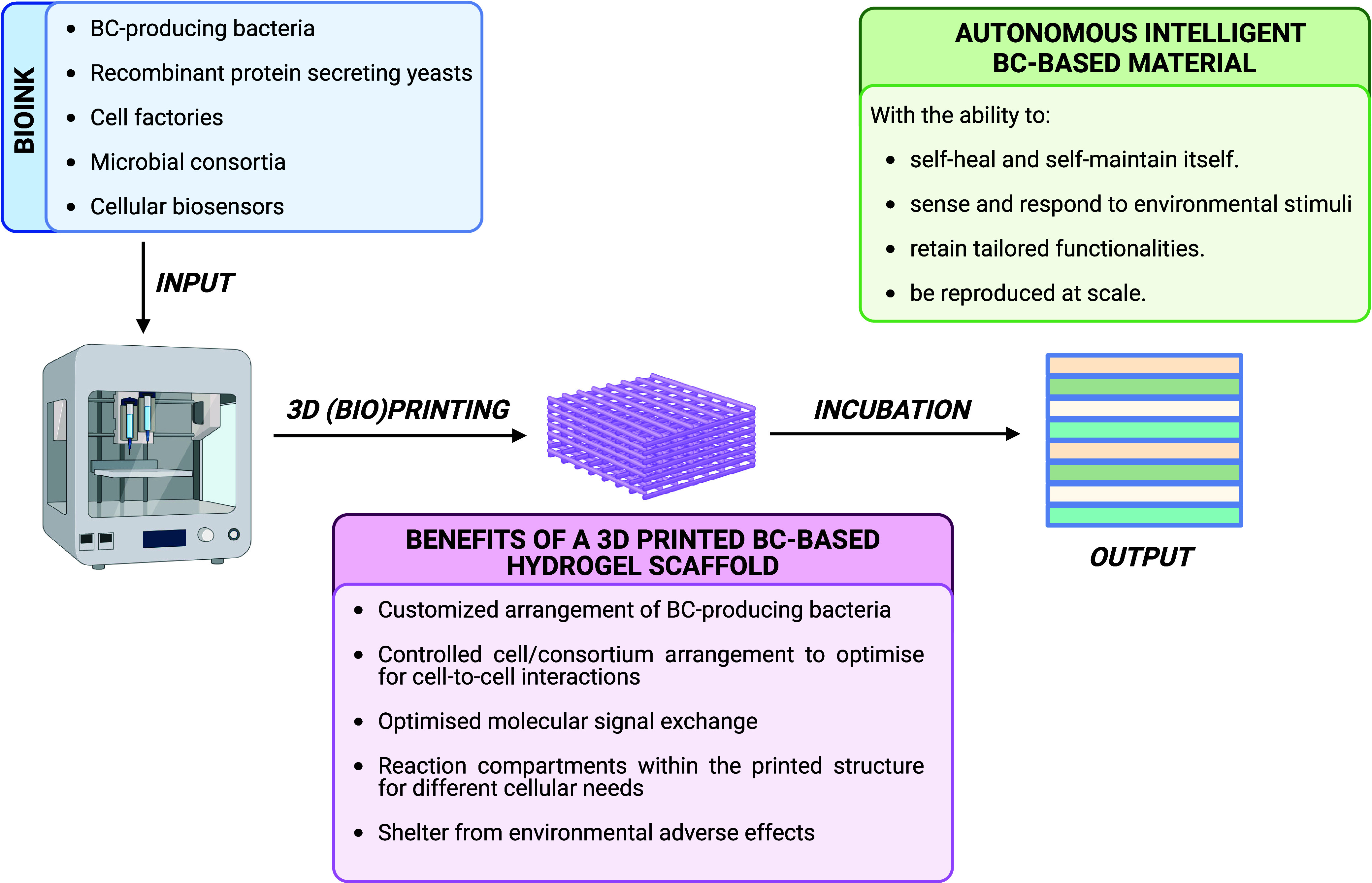
3D Bioprinting Process: bioink, containing BC producers and genetically engineered organisms, is used as an input for the 3D bioprinter. The printed BC-based material is then incubated to allow the growth of active biological elements, resulting in a tailored, autonomous intelligent material that is self-healing and responsive.

A second consideration is to ensure that cells within the 3D printed matrix are stable and have sufficient access to nutrients and oxygen, as well as being able to differentiate. Cell-laden hydrogels are only useful if they lead to bioactive 3D-printed cell structures,^146^ and a dense 3D-printed matrix cannot support the oxygen needs of aerobic cell processes such as BC synthesis.^147^ Indeed, Schaffner *et al.* (2017) reported varying BC densities within the facial skin scaffold that correlated to oxygen availability.^148^ This challenge can be overcome with microbial consortia. For instance, through co-culturing BC-producing *Acetobacter aceti* with photosynthetic microalgae *Chlamydomonas reinhardtii*, cellulose synthesis can be expanded beyond the air–water interface throughout the culture vessel.^149,150^ Once the challenge of providing sufficient oxygen to the 3D-printed matrix for effective BC synthesis is resolved, it will enable the production of homogeneous, dense BC-based materials. These BC-based ELMs will then be able to take full advantage of the spatiotemporal control offered by 3D printing combined with the cell enhancement capabilities provided by synthetic biology. Figure 6 summarizes the benefits of 3D printing BC-producing bacteria in conjunction with other cellular tools engineered by synthetic biology.

3D-printed BC-based materials that are dense and scalable could be incorporated into more general research efforts revolving around BC-based biomaterials for industrial applications. Such 3D printed materials are being investigated due to their ability to convert CO_2_ into oxygen; the incorporation of microalgae into 3D printed biomaterials designed to act as absorbing and releasing systems are particularly relevant for fields such as wound healing and regenerative medicine, biosensors, and urban construction.^141,146,151,152^ In addition, 3D bioprinting is being investigated as a potential technology for making astronaut skin transplants that can sense and respond to different conditions in space as well as a bioregenerative life support system where waste and CO_2_ produced by astronauts can be processed and recycled into oxygen and other useful metabolites.^142^

Furthermore, 3D bioprinting enables the construction of hierarchically structured materials containing cell arrangements that optimize cell-to-cell interactions in ways that cannot be achieved through liquid cocultures.^144,153^ For example, 3D bioprinting could enable the spatial segregation of strains in a consortium to minimize the consortium instability that results from nutrient competition.^154^ In the context of BC-based materials, the use of 3D bioprinting to segregate BC-producing and BC functionalizing microbes in a community could enhance ELM material yield and efficiency of functionalization.^141^ Furthermore, 3D bioprinting offers the potential of creating reaction compartments within the printed structure that cater to different cellular needs (e.g., optimal pH for an enzymatic activity to construct selectively active functional materials), bypassing the need to optimize a coculture media that compromises individual strain growth in favor of the consortium.^142^

The control of spatial cell arrangement offered by 3D bioprinting is an unrivaled benefit of the technology that could aid construction of highly customized ELMs with standardized features, leading to increased efficiency in the ELM research field.

## Translation into Industry

The natural properties of BC produced by wild-type *Komagataeibacter* strains have been leveraged by several industry leaders in biopolymer synthesis and pharmaceuticals over the past few decades, and BC now has widespread use in the biomedical industry, particularly for biocompatible wound dressings, burn dressings, and cosmetic face masks. The customizable properties of BC have also driven interest in other industries ranging from food, textiles, battery, paper, and composites manufacturing as listed in Table 2.

**Table 2:** Usage of BC across various industries by different companies

Primary Industry	Companies
Biomedical or Cosmetic	Evonik (JeNaCell acquisition; Germany), Bowil Biotech (Poland), DePuy Synthes (Johnson & Johnson; USA), KKF Polymers (Germany), Hylomorph (Switzerland), SK Bioland (Korea), Cellink Bioprinting AG (Sweden) Xylos Corporation (USA), fzmb GmbH (Germany), Innovatec (Brazil), Lohmann & Rauscher (Germany), Axcelon Biopolymers Corp (Canada), S2M medical (Sweden), AxCell Laboratories (Canada) and -commercial enterprises across the world
Food	BIOWEG UG (Germany), Satisfibre (Portugal), and commercial enterprises across the world
Textile	Polybion (Mexico), Nanollose (Australia), Gozen Institute (Türkiye), Next Gen Shoes (Spain),
Composite	Malai (India), Modern Synthesis (UK), Rheom Materials (USA), Cellugy (Denmark), Symmetry Wood (USA), and commercial enterprises across the world
Battery	Samsung Electronics (Korea)
Production	CP Kelco (USA), Kusano Sakko (Japan), and commercial enterprises across the world

As the industry has grown, genetic engineering research has focused on developing proprietary strains optimized for increased BC production. Some of these efforts have led to published papers, but much of the key work is described in patents. Notable examples have engineered BC bacteria to increase cellulose production yields, such as deletion of insertion sites in *bcsA* or overexpression of *bcsD* to change cellulose crystallinity.^155,156^ Other more recent examples have included engineering the same bacteria to also express therapeutic compounds and pigments, or to metabolize specific substrates.^157−159^

Co-culturing strategies, which often involve engineering bacteria or yeast alongside BC-producing organisms, have also been patented to facilitate the expression of functional proteins fused to CBMs, or using the microorganism as a live therapeutic, such as coculturing with *Lactobacillus rhamnosus* or *L. plantarum* for balancing *Staphylococcus aureus* populations in the skin microbiome or to form a secondary coating or coloration.^160−164^ These strategies create multifunctional BC-based materials integrating therapeutic, antimicrobial, or bioactive properties directly into the cellulose matrix.

A significant challenge faced by industrial BC manufacturers is production scale-up and batch standardization, which rival commercial viability as the biggest concerns. To address this, innovations in the development of bioprocesses that allow for the growth of BC in customizable dimensions have been described. For instance, the Horizontal Lift reactor developed by JeNaCell allows for the continuous production of BC sheets, while the Matrix Reservoir technology by KKF Polymers enables the layer-by-layer formation of hollow BC tubes for vascular grafts or implants.^23,26,165,166^ In structural customization, Hylomorph has developed BC pouches to encase implants with microstructure indentation designed to limit scar tissue formation.^167−169^ Furthermore, a nanocellulose-based 3D bioprinting bioink by Cellink Bioprinting has for both nonliving and living, 3D printed scaffolds for tissue engineering and regenerative medicine.^170^

Variability in BC production often dictates which strains can be used for large-scale manufacturing while remaining cost-effective. To successfully develop genetically engineered *Komagataeibacter* strains, collaboration with industrial-scale production partners is essential.^169^ The modularity of SynBio toolkits is a major benefit for this, as they enable the introduction of complex genetic circuits into different strains and organisms. There is still much to be explored in the SynBio design landscape to understand what determines industrial success. Is the gene stability improved by genome integration? Which selectable markers best maintain population stability? What external inducers can modulate the expression of metabolically intensive compounds at scale? And can co-culture approaches be scaled effectively into industry?

## Conclusion and Future Perspectives

The convergence of SynBio and material science opens many possibilities for designing and developing a new generation of smart materials that are sustainable, adaptable, flexible, and capable of sensing and responding to environmental stimuli. BC, with its significant structural and physical advantages and ability to be microbially synthesized at high yields, stands out as a significant player in this field. Recent advances now make it possible to use modular cloning to engineer BC-producing bacteria, particularly species of *Komagataeibacter*, such as *K. xylinus*, *K. hansenii*, and *K. rhaeticus*.

While much of the research on genetic manipulation of BC producers has focused on enhancing BC production yield, there have been efforts to tailor BC properties for specific applications. These include producing therapeutically important copolymers and creating colored BC as sustainable alternatives to traditional textiles. However, when it comes to responsive and adaptable materials, ELMs containing functionalized organisms or biological factors hold significant potential. SynBio offers a wide range of opportunities, allowing ELMs to respond effectively to chemical stimuli, including human hormones and drug molecules, as well as to light. Additionally, physical properties, such as the stiffness or degradability of BC, can be finely tuned by using genetically engineered organisms. The studies described in this review are examples that highlight the potential of engineered BC-based materials and ELMs for various applications, from sustainable product design to bioremediation.

Despite the advantages and possibilities offered by BC-based materials, challenges remain. Ensuring long-term cell viability and activity within ELMs is also an unsolved problem, and maintaining optimal conditions such as nutrient and water content within ELMs is a significant challenge. There are also limited tools for genome engineering in BC producers, and genetic engineering in these bacteria is generally low efficiency and outcomes are often hard to predict. Developing more specialized genetic tools and methodologies tailored for specific applications could provide solutions. In this regard, more accurate and comprehensive genome-scale metabolic models could offer valuable system-level insights into the intracellular mechanisms of BC producers. Such models could lead to the development of tailored strains capable of faster growth under optimized conditions or ones able to utilize inexpensive carbon sources for cellulose synthesis, thereby enabling media optimization for various needs.

While synthetic and systems biology tools and techniques are indispensable for BC-based biomaterial production, advances in fabrication techniques could also address many bottlenecks associated with ELMs. 3D bioprinting, a relatively new approach to manufacturing living materials and systems, could be an effective solution to current challenges, particularly those related to ELMs, such as irregular microenvironments within the material or the random distribution of organisms. Through 3D bioprinting, ELMs could be compartmentalized in a manner similar to natural systems, and 3D-modeled fabrication could provide more predictable behaviors of BC-based ELMs.

BC produced by *Komagataeibacter* is also being studied for potential applications in space and microgravity environments, such as the International Space Station.^171^ This suggests that BC-based materials may find use beyond Earth, where they originated. With the resolution of key bottlenecks, high-value, effective, and long-lasting smart living devices can be designed and developed soon. These living materials could also be utilized in biomedical applications, wherever they meet regulations. Undoubtedly, advances in SynBio will continue to drive the design of BC-based materials and the development of ELMs, leading to more innovative solutions to many of the challenges faced in the modern world.

## Supporting Information Available

**Figure S1**: Taxonomic relationships of cellulose-producing bacteria (PDF)
