# Supplementary material for: Modulating Microbial Materials - Engineering Bacterial Cellulose with Synthetic Biology
Source: ACS Synth Biol. 2024 Nov 7;13(12):3857–75. doi: 10.1021/acssynbio.4c00615 (PMC11669176; doi:10.1021/acssynbio.4c00615)
Supplement: Supplementary file 1 — sb4c00615_si_001.pdf [file sb4c00615_si_001.pdf]

SUPPORTING INFORMATION

Modulating Microbial Materials - Engineering Bacterial Cellulose with Synthetic Biology

Koray Malcı[a,b], Ivy S. Li[a,b], Natasha Kisseroudis[b,c], Tom Ellis[a,b,*]

[a] Department of Bioengineering, Imperial College London, London SW7 2AZ, UK

[b] Imperial College Centre for Synthetic Biology, Imperial College London, London SW7 2AZ, UK

[c] Department of Life Sciences, Imperial College London, London SW7 2AZ, UK

[*] Corresponding author: t.ellis@imperial.ac.uk

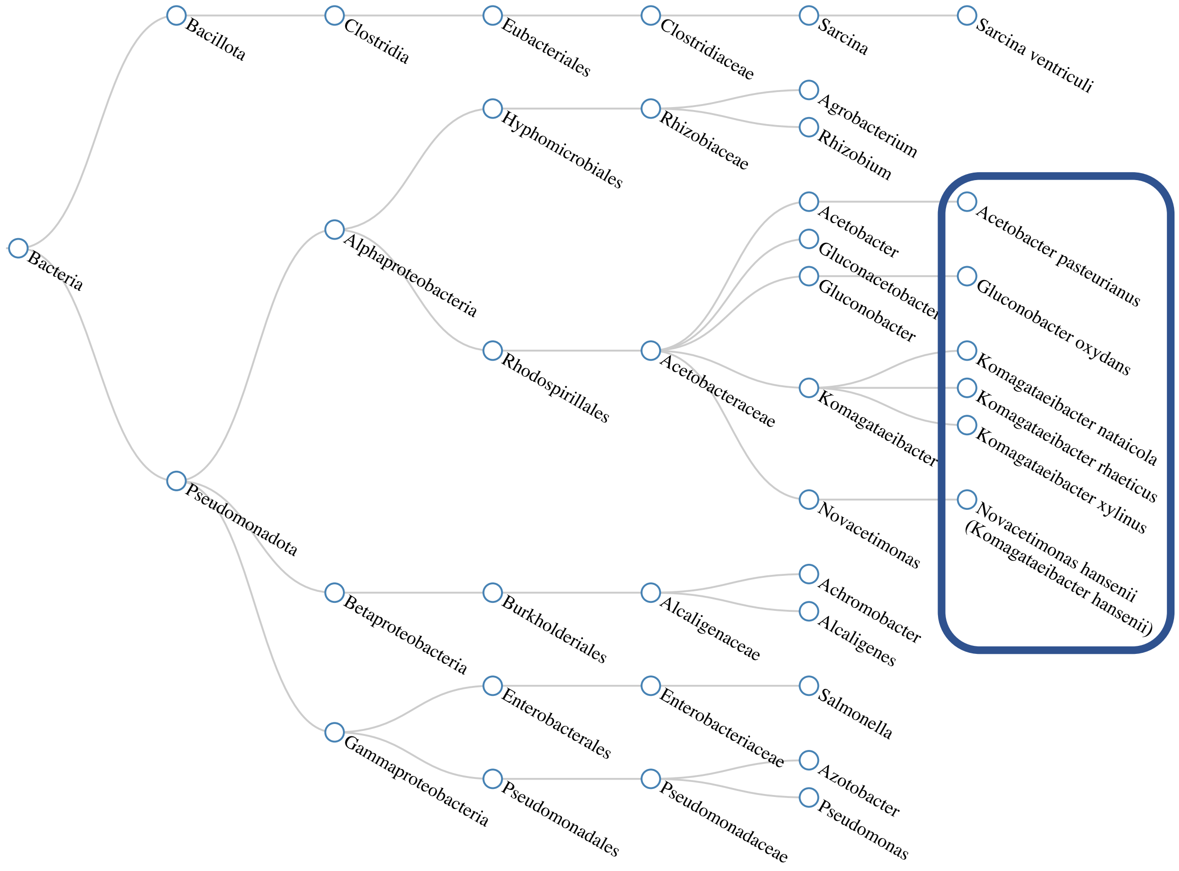

Figure S1: Taxonomic relationships of cellulose-producing bacteria. Except for *Sarcina ventriculi*, all BC producers are Gram-negative bacteria. BC-producing species within the *Acetobacteraceae* family, which are the focus of this review, are highlighted in the blue box. The taxonomic tree was constructed using TaxaIlnomy [1]

References

(1) Sakamoto, T.; Ortega, J. M. TaxaIlnomy: An Extension of NCBI Taxonomy That Produces a Hierarchically Complete Taxonomic Tree. *BMC Bioinformatics* 2021, *22* (1), 388. https://doi.org/10.1186/s12859-021-04304-3.
